# Chronic Organic Solvent Exposure Changes Visual Tracking in Men and Women

**DOI:** 10.3389/fnins.2017.00666

**Published:** 2017-11-30

**Authors:** Ana R. de Oliveira, Armindo de Arruda Campos Neto, Paloma C. Bezerra de Medeiros, Michael J. O. de Andrade, Natanael A. dos Santos

**Affiliations:** ^1^Department of Psychology, Federal University of Paraíba, João Pessoa, Brazil; ^2^Department of Electro-Electronics, Federal Institute of Mato Grosso, Cuiabá, Brazil; ^3^Department of Psychology, Federal University of Piauí, Parnaíba, Brazil

**Keywords:** organic solvents, eye movement, neurotoxicity, visual processing, sexes

## Abstract

Organic solvents can change CNS sensory and motor function. Eye-movement analyses can be important tools when investigating the neurotoxic changes that result from chronic organic solvent exposure. The current research measured the eye-movement patterns of men and women with and without histories of chronic organic solvent exposure. A total of 44 volunteers between 18 and 41 years old participated in this study; 22 were men (11 exposed and 11 controls), and 22 were women (11 exposed and 11 controls). Eye movement was evaluated using a 250-Hz High-Speed Video Eye Tracker Toolbox (Cambridge Research Systems) via an image of a maze. Specific body indices of exposed and non-exposed men and women were measured with an Inbody 720 to determine whether the differences in eye-movement patterns were associated with body composition. The data were analyzed using IBM SPSS Statistics version 20.0.0. The results indicated that exposed adults showed significantly more fixations (*t* = 3.82; *p* = 0.001; *r* = 0.51) and longer fixations (*t* = 4.27; *p* = 0.001, *r* = 0.54) than their non-exposed counterparts. Comparisons within men (e.g., exposed and non-exposed) showed significant differences in the number of fixations (*t* = 2.21; *p* = 0.04; *r* = 0.20) and duration of fixations (*t* = 3.29; *p* = 0.001; *r* = 0.35). The same was true for exposed vs. non-exposed women, who showed significant differences in the number of fixations (*t* = 3.10; *p* = 0.001; *r* = 0.32) and fixation durations (*t* = 2.76; *p* = 0.01; *r* = 0.28). However, the results did not show significant differences between exposed women and men in the number and duration of fixations. No correlations were found between eye-movement pattern and body composition measures (*p* > 0.05). These results suggest that chronic organic solvent exposure affects eye movements, regardless of sex and body composition, and that eye tracking contributes to the investigation of the visual information processing disorders acquired by workers exposed to organic solvents.

## Introduction

Organic solvents are liquid hydrocarbon fractions derived from petroleum processing. They contain carbon and hydrogen atoms ranging from about C5 to C20 that have boiling points of approximately 35–370°C (Mckee et al., 2015). Many hydrocarbons have complex and variable compositions but most share similar toxicological properties (Kamal et al., 2012). Compounds of benzene, toluene, ethylbenzene, and xylene (known as BTEX) are potentially toxic to human health because they are volatile and lipophilic.

Organic solvent absorption can occur via inhalation or through the skin or eyes (Kamal et al., 2012). These solvents are easily dissolved by the body and distributed in lipid-rich organs such as the brain; as such, they can penetrate the hematoencephalic barrier (Dick, 2006). Thus, solvents can damage the axonal myelin sheath and cell membrane (Niklasson et al., 1993; Boeckelmann and Pfister, 2003), which can affect central nervous system (CNS) structure and function (Barbosa et al., 2015).

Many studies have suggested that CNS sensory systems and motor functions (Dick, 2006) are vulnerable to toxic insult (Klaassen and Watkins, 2012). Much research has shown that the visual system, likely because it is one of the most studied systems, is highly affected; in many cases, visual changes occur in the absence of clinical signs of toxicity (Fox, 2015).

Organic solvents usually affect certain visual structures such as the rods and cones, the lateral geniculate nucleus (LGN), and the visual cortex, changing the macula and retina, decreasing corneal sensitivity, and increasing lens opacification (Fox, 2015) as well as disrupting ion channels and neurotransmitter receptors (Bowen et al., 2006). Solvents can also cause functional changes in color vision, achromatic contrast sensitivity in both the visual (Costa et al., 2012; Lacerda et al., 2012) and oculomotor (Baloh et al., 1979; Spivey et al., 1980; Specchio et al., 1981; Ödkvist et al., 1987; Niklasson et al., 1993; Herpin et al., 2008) fields.

Eye movements might be important when investigating CNS changes because many cortical and subcortical regions are involved in visual processing and the coordination of these movements (Duchowski, 2007). Eye control is characterized by processes such as saccades and fixations that enable the orientation and stabilization of sight (Herpin et al., 2008). Eye orientation involves saccadic and smooth pursuit eye movements, whereas eye stabilization works through eye reflexes of vestibular and visual origin (Herpin et al., 2008).

Saccades and fixations are among the most known and studied eye movements because they possess measurable dynamic properties (Netto and Colafêmina, 2010). Saccades are rapid eye movements that occur at angular velocities of up to 900 degrees per second that are used to reposition the fovea at points of interest and build an understanding of the visual environment (van Zoest and Hunt, 2011). Fixations are the stopping points between saccades where the eyes pause to capture scene-specific information (Duchowski, 2007). The observation of a visual scene normally involves approximately three fixations per second (Goldstein, 2010), and the period between each fixation lasts a few 100 ms. Saccades take approximately 40 ms and redirect the center of vision of the visual field (Kandel et al., 2014).

The magnitude and direction of saccadic eye movements can be modified voluntarily; however, their speed usually only changes in cases of fatigue, the use of psychoactive drugs, illness, or injury (Kandel et al., 2014). According to Kandel et al., bilateral lesions of the frontal eye fields and superior colliculus render monkeys incapable eye movement.

An analysis of eye movements (e.g., fixations) can help to detect changes before neurological damage occurs or identify a specific eye-movement pattern that characterizes a visual defect (Fox, 2015). In this regard, previous studies have evaluated the eye-movement patterns of men exposed or not exposed to lead (Baloh et al., 1979; Spivey et al., 1980; Specchio et al., 1981). The results demonstrated longer fixation durations and slower speeds as well as slower speed and mean saccade precision in the group exposed to this heavy metal (Baloh et al., 1979; Spivey et al., 1980). Specchio et al. (1981) found abnormalities in the smooth eye pursuit movement system of the group exposed to lead.

Niklasson et al. (1993) developed an experimental animal model based on the vestibulo- and opto-oculomotor system (VOOM) to measure the effect of toluene, styrene, trichlorethylene, and trichloroethane on the sensorimotor integration properties in the central part of the vestibular system of male and female rats. These authors observed that animals exposed to toluene, styrene, and trichlorethylene were unable to maintain an eye position toward the first position until another saccade was emitted, whereas exposure to trichloroethane did not produce the same result. Niklasson et al. concluded that the cerebellar-vestibular circuit is a common site of action but that different solvents have different effects upon it.

Herpin et al. (2008) used posturography and oculomotricity tests to evaluate the oculomotor function of hospital laboratory technicians (*n* = 12) who had been exposed to low levels of organic solvents (toluene, ethanol, formaldehyde, and paraffin) for an average of 24 years and compared their performance with women who had not been exposed to these solvents (*n* = 12). The exposed workers showed a reduced ability to resolve sensory conflict situations and increased saccadic reaction time. The authors reported that chronic exposure to low concentrations of organic solvents affect the vestibular pathways involved in the control of posture and the control of oculomotor response.

Hunting et al. (1991) evaluated the risk factors associated with the occurrence of slips, trips, and falls (STFs) in painters (*n* = 123) to test the assumption that organic solvent exposure is positively associated with the risk of these accidents. Participants performed tests in the laboratory and answered questionnaires each week to monitor both STFs and exposure indices. The study lasted 11 months. The results suggested that in the weeks during which the level of organic solvent exposure increased, a greater number of STFs occurred. The authors attributed these results to inefficient eye-movement performance. STFs might be related to changes in cerebral processing, particularly with regard to functions related to postural monitoring and control (Vouriot et al., 2004).

Most studies that have investigated the effects of organic solvent exposure have been conducted with men and have not use eye-movement tracking. We even found studies that evaluated the neurotoxic effects on the eye-tracking of men exposed to heavy metals such as lead (Baloh et al., 1979; Spivey et al., 1980; Ödkvist et al., 1987). Comparisons of men and women with regard to the effects of organic solvent exposure is important because the toxicokinetics of the solvents and toxic responses might affect people differently based on body composition (Iregren et al., 2002; Ernstgård et al., 2003; Gochfeld, 2007; Vahter et al., 2007; Weiss, 2011; Mergler, 2012).

Generally, women weigh less and have lower body mass indices (BMIs) as well as higher levels of body fat and organic blood flow than men (Klaassen and Watkins, 2012). Conversely, men have greater plasma volumes than women (Vahter et al., 2007). Solvents carried by the arterial blood are distributed according to the rate of tissue blood flow and the tissue/blood partition coefficient of the solvent (Klaassen and Watkins, 2012). Lipophilic solvents do not bind to plasma proteins or hemoglobin; rather, they are divided in hydrophobic sites in the molecules. They are distributed in the phospholipids, lipoproteins, and cholesterol in the blood (Klaassen and Watkins, 2012). This distribution of solvents has resulted in opposing views concerning the effects of organic solvents in men's and women's bodies. For example, Gleiter and Gundert-Remy (1996) argue that women are sensitive to the above effects because this sex has a higher percentage of body fat. Sato et al. (1991) demonstrated this finding 16 h after solvent exposure: women had an approximately 30% higher solvent concentration than men. On the other hand, Gandhi et al. (2004) as well as Soldin and Mattison (2009) suggested that women are less sensitive than men because body fat increases the distribution volume of lipophilic agents and these agents are metabolized more slowly in women. In this sense, changes in visual processing might be related to body composition, which differs between men and women (Vahter et al., 2007).

Subtle changes in visual processing can have immediate and long-term effects on an individual's mental and physical health and social functioning (Klaassen and Watkins, 2012). Cognitive changes also occur because certain mental representations are visual. According to Klaassen and Watkins (2012), it is important to detect signs of toxicity early because visual system losses can increase the risk of workplace accidents, generate high costs for medical and social services, and lead to lost productivity as well as a decrease in quality of life.

**Figure 1:**
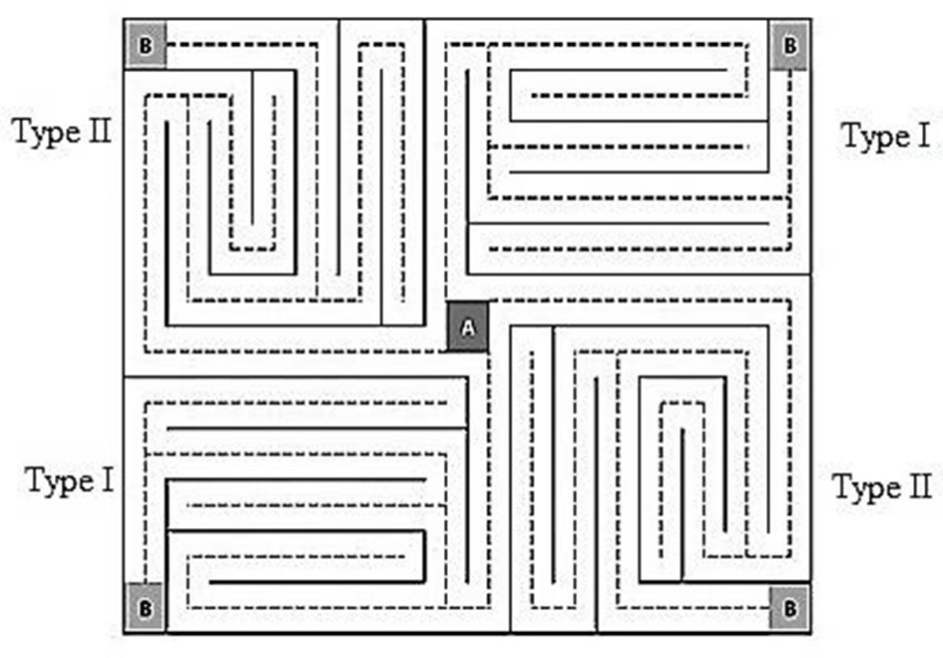
The VMT used in the eye-movement tracking task. The maze is a rectangle with a central starting point (point A) and four destination points (point B) at each of the four corners. Starting point A connects each of the four paths via independent dotted lines. Two paths exist: Type I and Type II (i.e., the upper left corner route is similar to the lower right corner route, and the lower left corner route is similar to the upper right corner route).

Therefore, the objectives of the current study were to record and compare the eye movements of men and women with and without a history of chronic organic solvent exposure as well as investigate whether differences exist in the tracking of a visual scene in the form of a maze (Figure 1) based on the number and duration of fixations to compare the performance of (1) all exposed vs. non-exposed participants, regardless of sex; (2) exposed vs. non-exposed men; (3) exposed vs. non-exposed women; and (4) exposed men vs. exposed women. If the main hypothesis was confirmed, then we planned to investigate whether the effects of chronic organic solvent exposure were sex-related, considering specific body composition indices (i.e., weight, fat mass, lean body mass, fat free mass, waist-hip ratio, visceral fat, and body water). This study was motivated by the lack of research investigating the effects of organic solvents on eye-movement patterns, particularly those comparing the effects between men and women. Our *a priori* hypotheses were that organic solvent exposure would change eye-movement patterns and that exposed men would have more and longer fixations when completing a task compared with women because of the differences in body composition between the sexes (Gandhi et al., 2004; Soldin and Mattison, 2009). Generally, women tend to have higher concentrations of fat, which might act as a protective factor, thereby preventing solvents from acting directly on the myelin sheaths of neurons.

## Materials and methods

### Participants

The participants included 44 volunteers, 18–41 years old, 22 of whom were men (11 exposed and 11 controls), and 22 of whom were women (11 exposed and 11 controls). The participants were assigned to the above four groups. Table 1 summarizes the sociodemographic characteristics of the groups as well as shows the mean and standard deviation (SD) for the variables age, education, and length of service in years.

**Table 1:** Sociodemographic data of the EMG, MCG, EFG, and FCG.

**Variable**	EMG^*^ (*n* = 11)		EFG^**^ (*n* = 11)		MCG^***^ (*n* = 11)		FCG^****^ (*n* = 11)	
	**Mean**	***SD***	**Mean**	***SD***	**Mean**	***SD***	**Mean**	***SD***
Age (years)	30.18	6.48	26.91	5.86	26.73	6.50	26.55	6.50
Education (years)	9.73	2.10	10.36	0.92	9.73	1.85	10.45	1.51
Exposure time (years)	6.53	4.38	4.70	3.53	^#^	^#^	^#^	^#^

* *Exposed male group*.

** *Exposed female group*.

*** *Male control group*.

**** *Female control group*.

*SD, standard deviation*.

# *Individuals with no exposure history*.

No significant between-group differences were observed in age (χ^2^ = 3.81; *p* = 0.28), education (χ^2^ = 1.42; *p* = 0.70), or exposure time (*U* = 47.5; *p* = 0.31) between exposed men and women. The working hours of the EMG and EFG participants were based on 8-h shifts/day, 6 days/week, with a 1-h interval for lunch. These participants worked with ethanol, gasoline, and diesel.

### Inclusion criteria

Participants who were employed 6 months or more as an gas station attendant (Campagna et al., 1995; Zaválic et al., 1998), had normal (20/20) or corrected visual acuity, were active during the morning or afternoon shift, and agreed to participate voluntarily in the study were included in the EMG or EFG. The MCG and FCG were composed of volunteers with normal or corrected visual acuity (20/20) who performed activities comparable with the skills required by service attendants. Table 2 shows the occupational activities performed by the control group participants, with the relative and absolute frequency of the participants.

**Table 2:** Occupational activities and number of participants in the MCG and FCG.

MCG^*^		FCG^**^	
Activity	*n*^..^	Activity	*n*^..^
Janitor	6	Janitor	5
General Services Assistant	2	Student	3
Student	2	General service assistant	2
Fiscal monitoring	1	Administrative assistant	1
Security guard	1	Day laborer	1
Total	12	Total	12

.. *Absolute frequency*.

* *Male control group*.

** *Female control group*.

### Exclusion criteria

Participants who had been exposed to chemical vapors in previous occupations (Semple et al., 2000); who had eye diseases, diabetes, or hypertension; and those who wore protective masks or goggles were excluded from the exposed groups. Participants who had histories of exposure to organic solvent vapors were excluded from the control groups. Furthermore, participants with ophthalmic impairments or strabismus, those who had received neurotoxic drug treatments over the past 6 months, and those with cerebrovascular disease (Lee et al., 2007, 2013) were excluded from all groups. Those practicing regular physical activity were also excluded because blood flow to the liver and kidneys decreases with exercise, which can decrease the biotransformation of metabolized solvents and urinary elimination (Klaassen and Watkins, 2012). Regular exercise was considered as a physical activity of 30 min, three or more times a week (Lee et al., 2009).

A total of 50 participants were initially recruited; however, six were excluded for the following reasons: four attendants (two women) reported regular physical activity; one man in the control group had amblyopia, and another had previously worked with paint.

### Apparatus

A 250-Hz High-Speed Eye Tracker Video Toolbox (Cambridge Research Systems; CRS, Rochester, Kent, England; http://www.crsltd.com/tools-for-vision-science/eye-tracking/high-speed-video-eye-tracker-toolbox/) monocular eye-movement tracker was used to measure eye fixation (brief stops). This tracker was connected to a Dell Precision T3500 computer with a W3530 graphics card and a Visual Stimulus Generator (*ViSaGe*; CRS) connected to two monitors: a flatscreen 19″ LG CRT video monitor used for stimulus presentation and a 21″ Dell LCT Precision T3500 monitor with a W3530 video card used to monitor the experiment through a mirrored screen. The LG CRT monitor's luminance was calibrated using an OptiCAL CRS photometer (average luminance, 34.4 cd/m^2^ and maximum luminance, 70.42 cd/m^2^). The accuracy of the equipment was provided through calibration. Additionally, the eye tracker ha 3-bit digital market input to record precise stimulus timing information directly from the ViSaGe (visual stimulus generator). Fixings were detected automatically using the software provided by the manufacturer of the eye tracker.

A chin and forehead support was used to maintain a distance of 570 mm between the participant and monitor, corresponding to one cycle per degree of visual angle (Corballis et al., 2002). The linear size horizontally and vertically is 17.06 cm. It was measured from the beginning of the monitor to the edge of the monitor, which is 17 degree of the visual angle since one degree is equivalent to 570 mm of dislocation (Corballis et al., 2002).

The High-Speed Video Eye Tracker Toolbox provides individual parameters for the cornea and the eyeball diameters that enable increased the accuracy of eye-rotation measurement (Cambrigde Research Systems, 2016).

### Visual stimuli

We used the Visual Maze Test (VMT) proposed by Santos et al. (2014) in psychophysic tasks to measure eye-movement tracking. This test consists of a rectangular maze with a central starting point (Point A) and four arrival points (point B), one in each of the four corners of the maze such that departure point “A” connects each of the four independent routes via dashed lines. The VMT has two different but equal paths (i.e., the upper left corner route is similar to the lower right corner route, and the lower left corner route is similar to the upper right corner route; Figure 1). This design enabled participants to choose a different route, when for whatever reason the test was not performed properly, thereby avoiding or decreasing the chance of a possible learning effect. Fixations occur when the eyes pause in a circular area with a radius of 20 mm for 100 ms, allowing specific scene information to be captured (Santos et al., 2014). The results shown represent the number and total duration of fixations that participants used while “going” through the maze from point A to point B.

The VMT was chosen to perform the eye-movement tracking (visual fixations) measurement because it is less affected by psychosocial factors and individual differences (e.g., education) than numbers, letters, or images with cognitive connotations. For more information about the maze, see Santos et al. (2014).

Matlab (Matrix Laboratory) version 7.2 was used to control the stimulus presentation and for data collection. Auxiliary software written in Java (“Eyetracker Translator”) was developed to extract the measures of interest (i.e., the number and duration of fixations).

### Body composition analysis

The Inbody 720 (MF-BIA8; Biospace Co. Ltd., Seoul, Korea) was used, which has an alternating current of 250 mA and a tetrapolar system with eight tactile electrodes at frequencies of 1, 5, 50, 250, 500, and 1,000 kHz. This equipment measures the change in body tissue impedance by sending detectable electrical signals from the body (Biospace, 2005). The system enables the segmental impedance of the upper and lower limbs and trunk to be measured for all frequencies. The body impedance scores are calculated by adding the segmental impedance values together (Lim et al., 2009).

The evaluation was performed according to the criteria specified in the equipment manual. Specifically, participants were instructed to (1) not perform physical activity the day before the evaluation; (2) have a meal 4 h prior to undergoing the examination; (3) use the bathroom minutes before the test (to reduce the volume of urine and feces); (4) remain standing for 5 min before the test; and (5) not wear metal jewelry or dental implants with metal. Lastly, women were excluded if they were menstruating. The participants' total weights, lean body mass indices (LBMs), and fat body mass (FM), waist-hip ratio (WHR), body water, visceral fat were used as body composition measurements. The duration of the evaluation was 10 min on average. This evaluation was carried out after the worker's shift.

### Procedure

Before starting the experiment, each participant performed a visual acuity test and answered a sociodemographic questionnaire. Only participants with normal acuity (6/6 or 20/20) or better in both eyes participated in this experiment. The dominant eye technique was then used to select one eye upon which to perform the experiment. For the dominant eye technique participants were set in front of a dark spot in the wall. After observing the point they were instructed to choose one eye at a time, and point at the dark spot. The open eye, which the participant was looking through when pointing to the spot more accurately was considered the dominant eye.

Each observer sat at the computer and positioned his or her head on a chin and forehead support. The observer's eye level was located 50 cm away from the center of the monitor. The steps involved in performing the test were eyetracker calibration (calibration, testing, and recording eye movement), maze presentation, and test completion. Calibration was necessary to synchronize the recording of the participants' eye fixations with the positions of the stimuli on the screen. Participants stared at the bright points displayed sequentially on the screen. After the last point was displayed, the experimenter repeated points that had not been calibrated. The maze was then displayed on the screen, and the participants were instructed to use their eyes to “go” from point A to any point B without leaving the dotted line. Eye movements were monitored continuously on the other monitor. When the participant had finished the task the session was closed. If the participant made a mistake, then the test was repeated, and the participant was invited to choose another maze route. In terms of the testing time, generally the study group took longest to comprehend and preform the task when compared to the control group.

The Ethics Committee of the Health Sciences Center (Centro de Ciências da Saúde; CCS) of the researchers' institution approved this study under registration number 21350113.9.0000.5188. Participants were informed of the private and confidential nature of their information and signed a Terms of Free and Informed Consent document in accordance with the Declaration of Helsinki.

### Statistical analyses

Data analysis was performed using IBM SPSS Statistics version 20.0.0 (IBM Corporation, Armonk, NY, USA). Data distribution normality was verified using the Kolmogorov-Smirnov and Shapiro-Wilk tests, and homogeneity of variance was verified using the Levene test. The data showed a normal distribution and homogeneity of variance. The *t-*test was used to examine the following between-group comparisons: (1) control vs. exposed; (2) exposed men vs. control men; (3) exposed vs. control women; and (4) exposed men vs. exposed women. Bivariate correlation analyses (Pearson's*r*) were also performed between the number and total duration of fixations and participant age, education, and exposure time as well as the physical measurements weight (kg), FM (kg), LBM (kg), fat free mass (kg), waist-hip ratio (WHR; cm^2^), visceral fat (cm^2^), and body water (*l*). Importantly, the dependent variable, number of fixations, was measured by counting its frequency, and the variable fixation duration was measured in seconds. The effect size was obtained using the following formula:

(1) $$\begin{array}{l} r=\sqrt{\frac{{\text{t}}^{2}}{{\text{t}}^{2}+\text{gl}}} \end{array}$$

For which:

*r* = effect size

t = difference between the means of the groups

gl = degrees of freedom.

## Results

**Figure 2:**
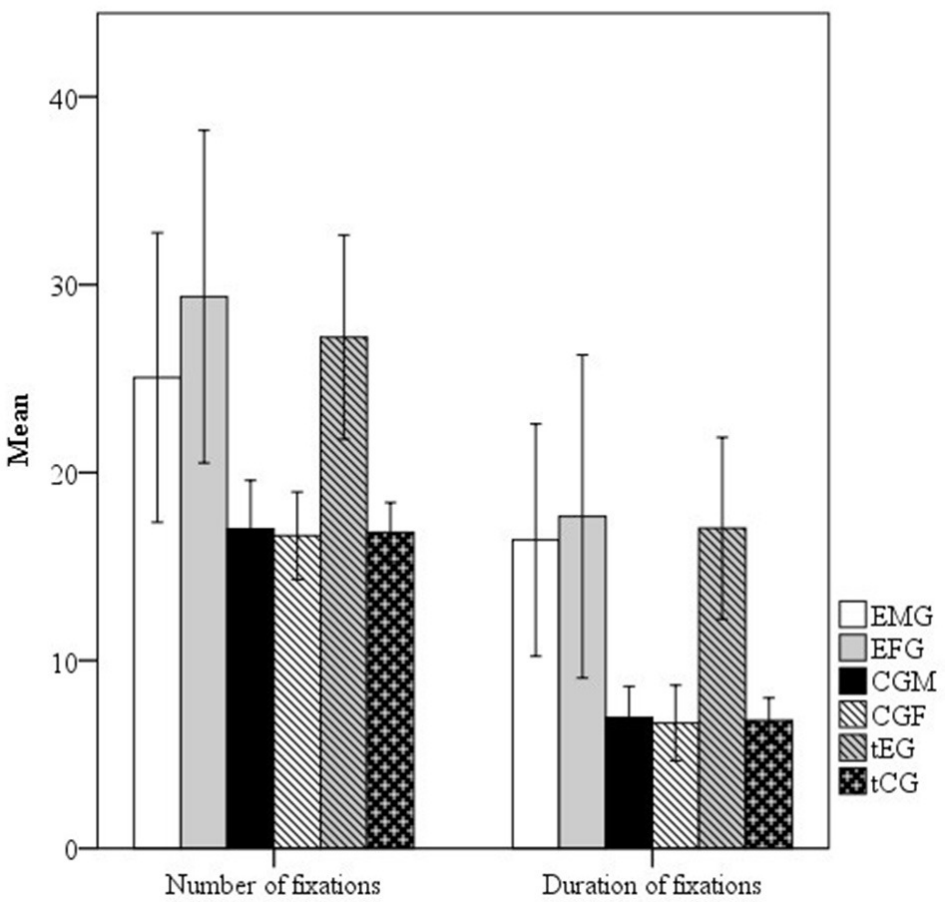
Mean and standard errors of the number of fixations and duration of fixations (in milliseconds) of the Exposed male group (EMG), Exposed female group (EFG), Control male group (CMG) and Control female group (CFG).

Descriptive statistics (means, confidence intervals, and SDs) for the number and total duration of fixation of the exposure groups (EMG and EFG) and control groups (MCG and FCG) are presented in the Figure 2.

The EMG and EFG generally had more number and total duration of fixations. The EFG had the most and longest fixations; these participants had on average 1.17 more fixations with an increase of 1.08 s in its total time than the EMG while traversing the maze from point A to point B.

Student's *t*-test was used to compare between-group performances and verify the presence of significant differences in number or total duration of fixation. Table 3 shows the results of these comparisons (*t*-test, *p*-value and effect size) between the exposed and control groups and their totals, i.e., total men and women exposed group (tEG) and total not exposed control group (tCG).

**Table 3:** The t-values, significance (*p*-value), and effect sizes associated with the comparisons of the mean number of fixations and duration of fixations between the tEG and tCG; EMG and EFG; EMG and CMG; and EFG and CFG.

**Variable**	**Comparison**	***t*-value**	***p*-value**	**Effect size**
Fixations	tEG vs. tCG	3.82	0.01^*^	0.51
	EMG vs. CMG	2.21	0.04^*^	0.20
	EFG vs. CFG	3.10	0.01^*^	0.32
	EMG vs. EFG	0.82	0.42	0.03
Duration	tEG vs. tCG	4.27	0.01^*^	0.54
	EMG vs. CMG	3.29	0.01^*^	0.35
	EFG vs. CFG	2.76	0.01^*^	0.28
	EMG vs. EFG	0.27	0.79	0.01

*tEG, Total exposed group*.

*tCG, Total control group*.

*EMG, Exposed male group*.

*EFG, Exposed female group*.

*CMG, Control male group*.

*CFG, Control female group*.

* *Significance, p < 0.005*.

The results in Figure 2, Table 3 show that compared with the MCG, the EMG had significantly more and longer fixations, with small and medium effect sizes, respectively. Compared with the FCG, the EFG also had a significantly more and longer fixations, with medium effect sizes. However, no significant differences (*p* > 0.05) were observed between the EFG and EMG (exposed women and men), demonstrating that exposed men and women showed similar eye-movement patterns.

Because age, education, and length of service as an attendant might affect the test results, correlation analyses were performed to evaluate the extent of these relationships. We did not find correlations between number of fixations and the age (*r* = 0.20, *p* = 0.37), education (*r* = −0.22, *p* = 0.32), or length of service (*r* = −0.16, *p* = 0.47) of the exposed groups as a whole. No correlations were found between the total duration of fixations and the age (*r* = 0.36, *p* = 0.10), education (*r* = −0.06, *p* = 0.79), or length of service (*r* = 0.03, *p* = 0.89) of the exposed groups as a whole.

**Figure 3:**
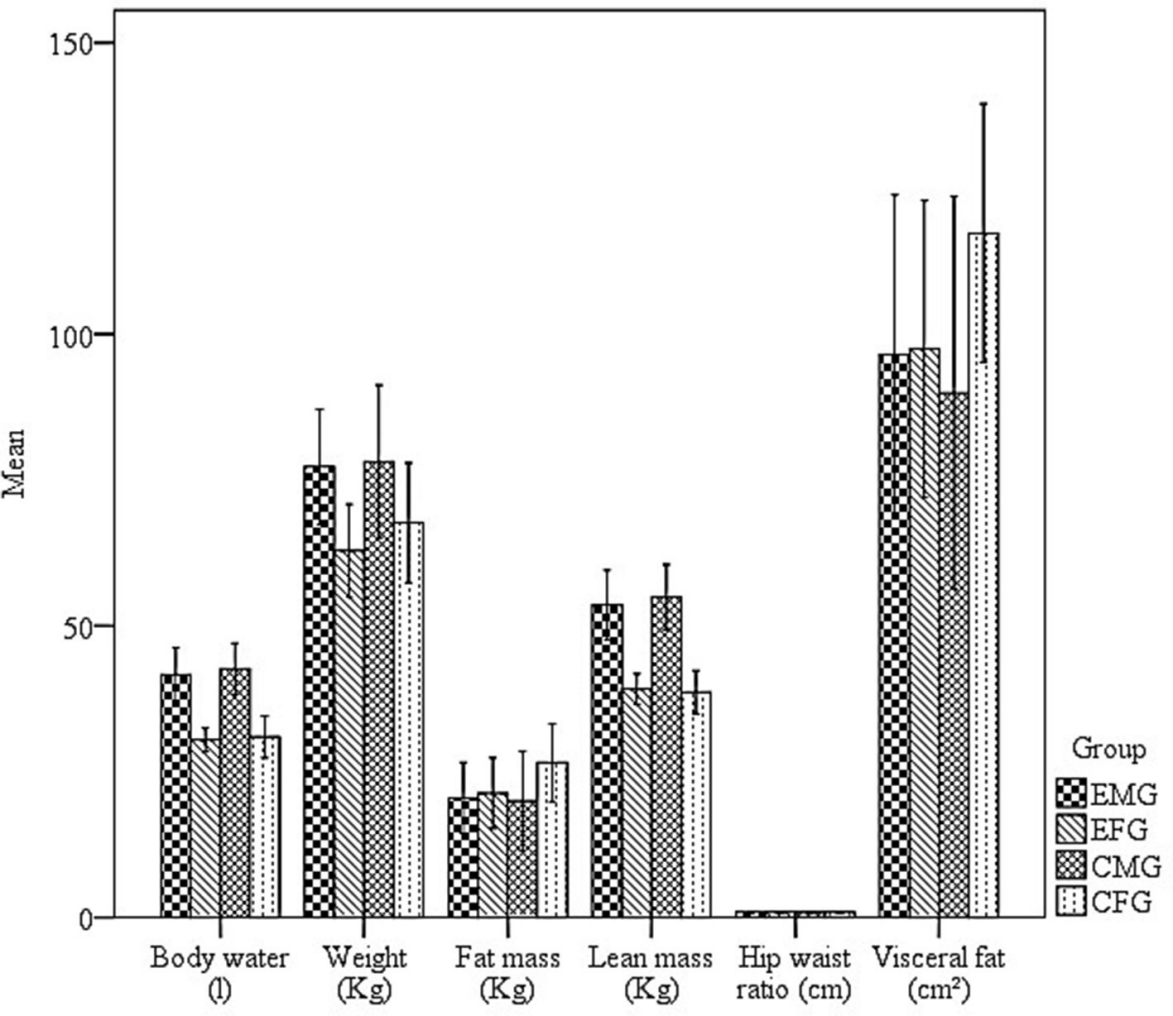
Mean and standard errors of the body water (*l*), weight (kg), fat mass (kg), lean mass (kg), hip waist ratio (cm), visceral fat (cm^2^), of the Exposed male group (EMG), Exposed female group (EFG), Control male group (CMG), and Control female group (CFG).

Another objective of this study was to test whether exposure to hydrocarbons would have a different effect on the body compositions of men and women. Figure 3 presents data relating to weight, fat mass, lean body mass, body water, fat free mass, WHR, and visceral fat for each group.

Figure 3 shows that the MCG had higher values for weight, LBM, fat free mass, and body water. The FCG had higher fat mass and visceral fat values, whereas the Waist-Hip Ratio (WHR) was similar between groups.

However, no correlations were found between the body composition indices and either the number or duration of fixations (*p* > 0.05), for either the exposed or control groups. This result suggests that the changes in the eye movements of participants exposed to organic solvents were not associated with body composition.

## Discussion

This study sought to measure the number and total duration of fixations of men and women with and without a history of chronic organic solvent exposure while following a visual route on a maze (Figure 1). The main goal was to investigate whether chronic organic solvent exposure affects eye-movement pattern. The secondary objective was to investigate whether the chronic effects of organic solvent exposure depend on sex or the differences between the body compositions of men and women. We hypothesized that chronic solvent exposure would affect eye movements and that these effects would be more pronounced in men than women because of their differences in body composition (Gochfeld, 2007; Vahter et al., 2007; Weiss, 2011; Mergler, 2012). Organic solvents are lipophilic compounds, and the higher concentrations of fat commonly found in women might function as a protective factor in the nervous system.

The main results confirm the principal hypothesis because chronic organic solvent exposure affected eye-movement pattern (i.e., the number and total duration of fixations). The secondary hypothesis was rejected because the effects of chronic organic solvent exposure occurred regardless of male/female body composition. In general, these results are new because previous studies have not evaluated the effects of hydrocarbon exposure on eye movements nor have they compared the results of men and women with regard to body composition as discussed below.

The results associated with the (tEG) included significantly more and longer fixations (*p* < 0.05) than those of the tCG (Figure 2, Table 3). That is, the tEG had 1.46 times more fixations and 2.50 times longer fixations than those of the tCG on average to “go” from point A to point B on the maze. Similar results were found for the EMG compared with the MCG as well as the EFG compared with the FCG. Both exposed men and exposed women had significantly more and longer fixations on average (*p* < 0.05) than non-exposed men and women (Figure 2, Table 3). Changes in the number and total duration of fixations related to hydrocarbon exposure were expected because previous studies using animal models have shown damage to the VOOM (Niklasson et al., 1993). Studies of humans have shown impairments in the visual system and basic visual functions such as visual contrast sensitivity to color and shape (Boeckelmann and Pfister, 2003; Costa et al., 2012; Lacerda et al., 2012).

It is not possible to compare past work with our study because it is one of the first to evaluate the effects of hydrocarbons on eye movements; furthermore, previous studies were performed with different models, populations, objectives, and methodologies (Baloh et al., 1979; Spivey et al., 1980; Specchio et al., 1981; Niklasson et al., 1993; Boeckelmann and Pfister, 2003; Gong et al., 2003; Costa et al., 2012; Lacerda et al., 2012; Kaur et al., 2014).

However, our results contribute to the literature investigating the neurotoxic effects of organic solvents on eye tracking and reinforce the hypothesis that solvents affect neural processing. One important question that deserves consideration is the following: Is it possible that organic solvent exposure does not affect the visual system or the nervous system diffusely? The answer might be “yes” because organic solvents are lipophilic compounds with a high affinity for organs and cellular structures such as myelin sheaths and cell membranes that are rich in fat (Dick, 2006) and can affect different CNS organs, structures, and functions. Importantly, the action mechanisms involved in the changes caused by organic solvent exposure are unclear, although the effects might depend on substance concentration and exposure time (Klaassen and Watkins, 2012). The literature also discusses the possibility that the brain damage related to organic solvent exposure might be due to the decrease in neurotrophic factor Serum B-cell lymphoma 2 (Bcl-2), which is derived from the brain, involved in cognitive function, and vulnerable to a variety of toxic effects (Hegazy et al., 2010). Furthermore, certain changes might be due to the damage of the cerebellar-vestibular function (Niklasson et al., 1993); however, the effects of different solvents in isolation should be investigated in animal models.

However, our results converge with the study of Ojanpää et al. (2006), because they can indicate not only deficts in the perceptual processes but also in the reduction of the neurocognitive processing speed, because the visual acuity was preserved. Ojanpää et al. (2006) verified ocular movements and performance in the task of visual search of individuals with and without Chronic Toxic Encephalopathy (CTE). The results showed that the visual search times of patients with CTE were longer, and they required considerably more ocular fixations than controls to find the target. Thus, the researchers believe that this reduction probably reflects a limited capacity for visual attention, since visual acuity and contrast sensitivity were preserved.

Another important finding of the present study was the non-confirmation of the secondary hypothesis; specifically, we did not find a difference between the EMG and the EFG (Figure 2, Table 3) with regard to the number or duration of fixations. Based on the literature, we expected that the effects of chronic organic solvent exposure would affect the eye movements of men and women differently given that such solvents are lipophilic compounds and differences exist in body composition by sex (Gochfeld, 2007; Vahter et al., 2007; Weiss, 2011; Mergler, 2012). Our *a priori* hypothesis was that the higher fat concentration usually found in women serves as a nervous system protective factor; however, our results did not support this conclusion. The Figure 2, Table 3 shows surprising results: The mean number (29.36) and duration (17.67 ms) of fixations of the EFG were higher than those (25.05 and 16.41 ms, respectively) of the EMG. Although these results were not significant (see comparison EMG vs. EFG, Figure 2, Table 3), they show that exposed women tended to perform slightly worse than exposed men. The results also failed to reveal relationships between the mean number and total duration of fixations and either body composition or exposure time (Figure 3). In general, these results do not support the hypothesis that women are less sensitive than men because body fat increases the distribution volume of the lipophilic agents that are metabolized more slowly in women (Gandhi et al., 2004; Soldin and Mattison, 2009). Although we did not find significant differences between exposed men and women, the results suggest that women can be more sensitive to solvent exposure than men. A possible explanation could be an association with their higher body fat percentage and higher internal solvent accumulation (Sato et al., 1991; Gleiter and Gundert-Remy, 1996; Löf and Johanson, 1998).

Given that the differences in the body compositions of men and women might influence the toxicokinetics of organic solvents (Vahter et al., 2007; Weiss, 2011), we also evaluated the relationships between the number and duration of fixations and the following body composition indicators: weight, water, lean body mass, fat mass, WHR, and visceral fat area (Figure 3). With regard to men, we expected that greater values of weight, lean mass, body water, and WHR would predict more and longer fixations. However, we found no correlation between the number and total duration of fixations and weight, lean mass, water, or WHR. With regard to women, we expected that greater fat mass and visceral fat areas would predict fewer and briefer fixations to reach the maze exit. Unexpectedly, no correlations were observed. In general, these results indicate that performance was independent of body composition and that fat might not be a protective factor against the effects of organic solvents.

The lack of a correlation between body composition indices and performance on the VMT might be attributed to several factors including (1) the small variation in the body and visceral fat indices of the current sample (Figure 3) and (2) hormonal differences given that previous studies have suggested that sex steroids are associated with visual processing (Abramov et al., 2012; Little, 2013; Demidova et al., 2014). For example, Demidova et al. (2014) noted that the brain-wave patterns of men viewing neutral color pictures displaying positive and negative emotions were similar to those of women during the follicular phase of the ovulatory cycle. Additional studies should evaluate whether any similarity exists in the eye-movement patterns of men and women depending on the ovulatory cycle phase of the latter.

In general, all of the results presented and discussed in this paper are consistent with the hypothesis that chronic organic solvent exposure changes neural activity and impairs performance on visual activities. These results support the use of the maze as an adequate stimulus to evaluate eye tracking. That is, the maze was resistant to psychosocial factors and individual differences because the effect of organic solvents did not interact with sex, age, length of service, or education. This null finding increases the possibility that the eye-movement changes occurred due to organic solvent exposure.

We suggest that future studies verify the relationships between performance on eye-tracking tests and low, medium, and high weight, fat, and lean body mass levels. Neurocognitive and behavioral tasks should be used to correlate with eye-movement tracking performance. Longitudinal studies evaluating eye tracking before worker exposure begins and at specific times (e.g., semi-annually and annually) might be useful in measuring the progression of the effect on eye movement. The current area of research is important because of the role that eye movements play in performing numerous activities of daily living. Oculomotor changes might lead to increased workplace accidents (Hunting et al., 1991) and consequently affect the general health and social lives of workers (Klaassen and Watkins, 2012).

## Author contributions

AdO, AC, and NdS contributed to the conception and design of the study. AdO and MdA collected the data with the high-speed video eye tracker. AdO analyzed and interpreted the data. AdO and PBdM wrote the article. NdS critically reviewed the study.

### Conflict of interest statement

The authors declare that the research was conducted in the absence of any commercial or financial relationships that could be construed as a potential conflict of interest.

## References

[B1] Abramov I. Gordon J. Feldman O. Chavarga A. (2012). Sex & vision I: spatio-temporal resolution. Biol. Sex Differ. 3, 1–14. 10.1186/2042-6410-3-20 22943466 PMC3447704

[B2] Baloh R. W. Spivey G. H. Brown C. P. Morgan D. Campion D. S. Browdy B. L.. (1979). Subclinical effects of chronic increased lead absorption—a prospective study. II. Results of baseline neurologic testing. J. Occup. Med. 21, 490–496. 469615

[B3] Barbosa I. A. J. Boon M. Y. Khuu S. K. (2015). Organic solvent exposure used in dry cleaning reduces low and high level visual function. PLoS ONE 10:e0121422 10.1371/journal.pone.0121422 25933026 PMC4416825

[B4] Biospace (2005). InBody 720 User's Manual. Available online at: http://www.bodyanalyse.no/docs/720%20users%20manual.pdf

[B5] Boeckelmann I. Pfister E. A. (2003). Influence of occupational exposure to organic solvent mixtures on contrast sensitivity in printers. J. Occup. Environ. Med. 45, 25–33. 10.1097/00043764-200301000-00009 12553176

[B6] Bowen S. E. Batis J. C. Paez-Martinez N. Cruz S. L. (2006). The last decade of solvent research in animal models of abuse: mechanist and behavioral studies. Neurotoxicol. Teratol. 28, 636–647. 10.1016/j.ntt.2006.09.005 17064879

[B7] Cambrigde Research Systems (2016). High-Speed Video Eye Tracker Toolbox. Available from: http://www.crsltd.com/tools-for-vision-science/eye-tracking/high-speed-video-eye-tracker-toolbox/

[B8] Campagna D. Mergler D. Huel G. Bélanger S. Truchon G. Ostiguy C.. (1995). Visual dysfunction among styrene-exposed workers. Scand. J. Work Environ. Health 21, 382–390. 10.5271/sjweh.53 8571095

[B9] Corballis P. M. Funnell M. G. Gazzaniga M. S. (2002). Hemispheric asymmetries for simple visual judgments in the split brain. Neuropsychologia 40, 401–410. 10.1016/S0028-3932(01)00100-2 11684173

[B10] Costa T. L. Barboni M. T. S. Moura A. L. A. Bonci D. M. O. Gualtieri M. de Lima Silveira L. C. (2012). Long-term occupational organic solvent exposure affects color vision, contrast sensitivity and visual fields. PLoS ONE 8:e42961 10.1371/journal.pone.0042961 PMC3419737 22916187

[B11] Demidova K. Y. Dubovik V. V. Kravchenko V. I. Makarchouk N. E. (2014). EEG activity during viewing of neutral and emotionally colored pictures by men and women with different levels of empathy. Neurophysiology 46, 160–168. 10.1007/s11062-014-9422-9

[B12] Dick F. D. (2006). Solvent neurotoxicity. Occup. Environ. Med. 63, 221–226. 10.1136/oem.2005.022400 16497867 PMC2078137

[B13] Duchowski A. T. (2007). Eye Tracking Methodology: Theory and Practice. London: Springer Verlag.

[B14] Ernstgård L. Sjögren B. Warholm M. Johanson G. (2003). Sex differences in the toxicokinetics of inhaled solvent vapors in humans 2. 2-propanol. Toxicol. Appl. Pharm. 193, 158–167. 10.1016/j.taap.2003.08.005 14644618

[B15] Fox D. A. (2015). Retinal and visual system: occupational and environmental toxicology, in Handbook of Clinical Neurology, eds Lotti M. Bleecker M. L. (Elsevier), 325–340. Available online at: http://www.sciencedirect.com/science/article/pii/B9780444626271000172 10.1016/B978-0-444-62627-1.00017-2 26563796

[B16] Gandhi M. Aweeka F. Greenblatt R. M. Blaschke T. F. (2004). Sex differences in pharmacokinetics and pharmacodynamics. Annu. Rev. Pharmacol. Toxicol. 44, 499–523. 10.1146/annurev.pharmtox.44.101802.121453 14744256

[B17] Gleiter C. H. Gundert-Remy U. (1996). Gender differences in pharmacokinetics. Eur. J. Drug Metab. Pharmacokinet. 2, 123–128. 10.1007/BF03190260 8839685

[B18] Gochfeld M. (2007). Framework for gender differences in human and animal toxicology. Environ. Res. 104, 4–21. 10.1016/j.envres.2005.12.005 16616135

[B19] Goldstein E. B. (2010). Sensation and Perception, 8th Edn. Belmont CA: Thomson Wadsworth.

[B20] Gong Y. Kishi R. Kasai S. Katakura Y. Fujiwara K. Umemura T.. (2003). Visual dysfunction in workers exposed to a mixture of organic solvents. NeuroToxicology 24, 703–710. 10.1016/S0161-813X(03)00034-2 12900083

[B21] Hegazy N. M. Gawad N. B. A. Metwally F. M. Ahmed H. H. Raouf E. R. A. Abrahim K. S. (2010). Neurotoxic effects of organic solvents in exposed workers: altered expression of some biochemical markers. N.Y. Sci. J. 3, 171–176. 10.7537/marsnys031110.25

[B22] Herpin G. Gauchard G. C. Vouriot A. Hannhart B. Barot A. Mur J.-M.. (2008). Impaired neuromotor functions in hospital laboratory workers exposed to low levels of organic solvents. Neurotoxicit. Res. 13, 185–196. 10.1007/BF03033502 18522898

[B23] Hunting K. L. Matanoski G. M. Larson M. Wolford R. (1991). Solvent exposure and the risk of slips, trips, and falls among painters. Am. J. Ind. Med. 20, 353–370. 10.1002/ajim.4700200308 1928112

[B24] Iregren A. Andersson M. Nylén P. (2002). Color vision and occupational chemical exposures: II. Visual functions in non-exposed subjects. NeuroToxicology 23, 735–745. 10.1016/S0161-813X(02)00113-4 12520763

[B25] Kamal A. Malik R. N. Fatima N. Rashid A. (2012). Chemical exposure in occupational settings and related health risks: a neglected area of research in Pakistan. Environ. Toxicol. Phar. 34, 46–58. 10.1016/j.etap.2012.02.009 22445870

[B26] Kandel E. Schwartz J. Jessell T. M. Siegelbaum S. Hudspeth A. J. (2014). Princí*pios de Neurociências* Porto Alegre: AMGH.

[B27] Kaur S. Azreena A. I. Leong K. N. Narayanasamy S. (2014). Effect of pesticides on color vision and anterior ocular structure of farmers. El Med. J. 2, 219–222. 10.18035/emj.v2i3.111

[B28] Klaassen C. D. Watkins J. B. III. (2012). Fundamentos em Toxicologia de Casarett e Doull. Porto Alegre: AMGH.

[B29] Lacerda E. M. C. B. Lima M. G. Rodrigues A. R. Teixeira C. E. C. de Lima L. J. B. Ventura D. F.. (2012). Psychophysical evaluation of achromatic and chromatic vision of workers chronically exposed to organic solvents. J. Environ. Public Health 2012:784390. 10.1155/2012/784390 22220188 PMC3246754

[B30] Lee E. H. Eum K. D. Cho S. I. Cheong H. K. Paek D. M. (2007). Acquired dyschromatopsia among petrochemical industry workers exposed to benzene. NeuroToxicology 28, 356–363. 10.1016/j.neuro.2006.05.005 16806479

[B31] Lee E. Paek H. D. Kho Y. L. Choi K. Chae H. J. (2013). Color vision impairments among shipyard workers exposed to mixed organic solvents, especially xylene. Neurotoxicol. Teratol. 37, 39–43. 10.1016/j.ntt.2013.02.005 23422509

[B32] Lee J. H. Kang W. Yaang S. R. Choy N. Lee C. R. (2009). Cohort study for the effect of chronic noise exposure on blood pressure among male workers in Busan, Korea. Am. J. Ind. Med. 52, 509–517. 10.1002/ajim.20692 19267371

[B33] Lim J. S. Hwang J. S. Lee J. A. Kim D. H. Park K. D. Jeong J. S.. (2009). Cross-calibration of multi-frequency bioelectrical impedance analysis with eight-point tactile electrodes and dual-energy X-ray absorptiometry for assessment of body composition in healthy children aged 6-18 years. Pediatr. Int. 51, 263–268. 10.1111/j.1442-200X.2008.02698.x 19405930

[B34] Little A. C. (2013). The influence of steroid sex hormones on the cognitive and emotional processing of visual stimuli in humans. Front. Neuroendocrin. 34, 315–328. 10.1016/j.yfrne.2013.07.009 23988462

[B35] Löf A. Johanson G. (1998). Toxicokinetics of organic solvents: a review of modifying factors. Crit. Rev. Toxicol. 28, 571–650. 10.1080/10408449891344272 9861527

[B36] Mckee R. H. Adenuga M. D. Carrillo J.-C. (2015). Characterization of the toxicological hazards of hydrocarbon solvents. Crc. Rev. Toxicol. 45, 273–365. 10.3109/10408444.2015.1016216 25868376

[B37] Mergler D. (2012). Neurotoxic exposures and effects: gender and sex matter! Hänninen lecture 2011. NeuroToxicology 33, 644–651. 10.1016/j.neuro.2012.05.009 22664101

[B38] Netto A. A. T. C. Colafêmina J. F. (2010). Saccadic Movements in subjects with cerebellar disorders. Braz. J. Otorhinolaryngol. 76, 51–58. 10.1590/S1808-86942010000100010 20339690 PMC9448920

[B39] Niklasson M. Tham R. Larsby B. Eriksson B. (1993). Effects of toluene, styrene, trichloroethylene, and trichloroethane on the vestibulo-and opto-oculo motor system in rats. Neurotoxicol. Teratol. 15, 327–334. 10.1016/0892-0362(93)90034-L 8277926

[B40] Ödkvist L. M. Arlinger S. D. Edling C. Larsby B. Bergholtz L. M. (1987). Audiological and vestibulo-oculomotor findings in workers exposed to solvents and jet fuel. Scand. Audiol. 16, 75–81. 10.3109/01050398709042159 3498206

[B41] Ojanpää H. Näsänen R. Päällysaho J. Akila R. Müller K. Kaukiainen A.. (2006). Visual search and eye movements in patients with chronic solvent-induced toxic encephalopathy. Neurotoxicology 27, 1013–1023. 10.1016/j.neuro.2006.04.009 16765447

[B42] Santos N. A. Campos Neto A. A. Sousa B. M. Pessoa E. D. C. Nogueira R. M. T. L. (2014). Matlab and eye-tracking: applications in psychophysics and basic psychological processes. Trends Psychol. 22, 589–601. 10.9788/TP2014.3-05

[B43] Sato A. Endoh K. Kaneko T. Johanson G. (1991). A simulation study of physiological factors affecting pharmacokinetic behaviour of organic solvent vapours. Br. J. Ind. Med. 48, 342–347. 10.1136/oem.48.5.342 2039747 PMC1012045

[B44] Semple S. Dick F. Osborne A. Cherrie J. W. Soutar A. Seaton A.. (2000). Impairment of colour vision in workers exposed to organic solvents. Occup. Environ. Med. 57, 582–587. 10.1136/oem.57.9.582 10935938 PMC1740009

[B45] Soldin O. P. Mattison D. R. (2009). Sex differences in pharmacokinetics and pharmacodynamics. Clin. Pharmacokinet. 48, 143–157. 10.2165/00003088-200948030-00001 19385708 PMC3644551

[B46] Specchio L. M. Bellomo R. Pozio G. Dicunonzo F. Assenato G. Frederici A.. (1981). Smooth pursuit eye movements among storage battery workers. Clin. Toxicol. 18, 1269–1276. 10.3109/00099308109035066 7341052

[B47] Spivey G. H. Baloh R. W. Brown C. P. Browdy B. L. Campion D. S. Valentine J. L.. (1980). Subclinical effects of chronic increased lead absorption-a prospective study. III. Neurologic findings at follow-up examination. J. Occup. Med. 22, 607–612. 7452384

[B48] Vahter M. Gochfeld M. Casati B. Thiruchelvam M. Falkfilippson A. Kavlock R.. (2007). Implications of gender differences for human health risk assessment and toxicology. Environ. Res. 104, 70–84. 10.1016/j.envres.2006.10.001 17098226

[B49] van Zoest W. Hunt A. R. (2011). Saccadic eye movements and perceptual judgments reveal a shared visual representation that is increasingly accurate over time. Vis. Res. 51, 111–119. 10.1016/j.visres.2010.10.013 20951719

[B50] Vouriot A. Gauchard G. C. Chau N. Benamghar L. Lepori M. L. Mur J. M.. (2004). Sensorial organisation favouring higher visual contribution is a risk factor of falls in an occupational setting. Neurosci. Res. 48, 239–247 10.1016/j.neures.2003.11.001 15154670

[B51] Weiss B. (2011). Same sex, no sex, and unaware sex in neurotoxicology. NeuroToxicology 32, 509–517. 10.1016/j.neuro.2010.09.005 20875453 PMC3044781

[B52] Zaválic M. Mandić Z. Turk R. Bogadi-Sare A. Plavec D. (1998). Quantitative assessment of color vision impairment in workers exposed to toluene. Am. J. Ind. Med. 3c3, 297–304. 10.1002/(SICI)1097-0274(199803)33:3<297::AID-AJIM12>3.0.CO2-V 9481429

